# Simulated Gastrointestinal Digestion of Tilapia (*Oreochromis* *niloticus*) Scale Hydrolysates Enhances ACE-Inhibitory Activity and Reveals Antioxidant Effects in STC-1 Cells

**DOI:** 10.3390/foods15142422

**Published:** 2026-07-08

**Authors:** Alisson Sisa, Mauricio Mosquera, Pablo Martín-Brieva, Paula Moreno-Ortega, Oscar Martínez Álvarez

**Affiliations:** 1 Department of Food Science and Biotechnology (DECAB), Escuela Politécnica Nacional, Quito P.O. Box 17-01-2759, Ecuador; alisson.sisa@epn.edu.ec; 2 Institute of Food Science, Technology and Nutrition (ICTAN-CSIC), 6th José Antonio Novais St., 28040 Madrid, Spain; pablombrieva@gmail.com (P.M.-B.); paula.moreno@ictan.csic.es (P.M.-O.); oscar.martinez@ictan.csic.es (O.M.Á.)

**Keywords:** tilapia scales, bioactive peptides, enzymatic hydrolysis, ACE-inhibiting activity, antioxidant effect, STC-1 cells, simulated gastrointestinal digestion, valorization, DPP-IV, prolyl endopeptidase

## Abstract

Tilapia (*Oreochromis niloticus*) scales represent an underutilized by-product with considerable potential as a source of bioactive peptides that can be released through enzymatic hydrolysis. This study evaluated the production of protein hydrolysates from demineralized tilapia scales using Alkaline Protease or Esperase 8.0 L^®^ (Alkaline Protease and Esperase 8.0 L® were kindly provided by Novozymes, Bagsværd, Denmark), and examined the impact of simulated gastrointestinal digestion (SGID) on their bioactivities. The highest degree of hydrolysis (DH) was obtained with Alkaline Protease (12.4%), generating peptide fractions predominantly below 1000 Da, with a major population around 888 Da. Both hydrolysates exhibited antioxidant activity, with the Alkaline Protease hydrolysate showing higher ferric reducing antioxidant power (FRAP) and Fe(II)-chelating activity. The hydrolysates also displayed significant angiotensin-converting enzyme (ACE) inhibitory activity (IC_50_: 13–14 µg/mL), dipeptidyl peptidase IV (DPP-IV) inhibitory activity (IC_50_: 0.66–0.69 mg/mL), and prolyl endopeptidase (PEP) inhibitory activity (IC_50_: 0.63–0.72 mg/mL). The digests were non-cytotoxic at the concentrations tested (<10 mg/mL) in STC-1 enteroendocrine cells. Following SGID, increased ACE-inhibitory activity was observed, with IC_50_ values as low as 4.6 µg/mL, whereas DPP-IV and PEP-inhibitory activities decreased. The intestinal digest of the Esperase hydrolysate also exhibited significant cellular antioxidant activity in the ROS assay. Overall, these results indicate that tilapia scale hydrolysates are a promising source of peptides associated with in vitro enzyme inhibitory and antioxidant activities. However, the specific bioactive peptides responsible for these effects were not identified. Therefore, further studies involving peptide characterization, bioavailability assessment, and in vivo validation are required to establish their physiological relevance and potential applications as functional ingredients.

## 1. Introduction

In recent years, the production of tilapia (*Oreochromis niloticus*) has reached approximately 4.5 million tons, positioning it as the third-most cultivated species globally, following carp and salmonids [1]. A significant portion of this production is allocated to industrial processing, resulting in the generation of substantial volumes of byproducts. Among these byproducts, scales constitute approximately 5–10% of the total fish weight and are estimated to produce up to ~50,000 tons of waste annually in certain contexts, thereby posing an environmental challenge if not managed appropriately [2]. Despite being commonly discarded, tilapia scales contain between 41% and 84% protein and are rich in amino acids such as Glu, Gly, Pro, Hyp, and Tyr, making them a promising raw material for obtaining high-value-added compounds [3]. However, current valorization remains limited and mainly focuses on gelatin extraction, which can present technological and sensory limitations, including undesirable odors. In this context, enzymatic hydrolysis has emerged as an efficient strategy for transforming collagen-rich residues into hydrolysates and peptide fractions with improved functional and biological properties [2].

Enzymatic hydrolysis is a well-established method for converting proteins into hydrolysates enriched with bioactive peptides. The application of commercial enzymes such as Trypsin, Pepsin, Flavourzyme, and Esperase facilitates the release of peptides with enhanced functional and nutritional properties [4]. In the fish industry, by-products may constitute between 50% and 80% of the total biomass, and hydrolysates derived from fish and side streams have been associated with various bioactivities, including antihypertensive, hypoglycemic, antioxidant, and antimicrobial effects [5]. Consequently, fish-derived bioactive peptides are being investigated as ingredients for preventive or complementary strategies in chronic disease management. These peptides generally exhibit milder mechanisms of action than synthetic drugs and are often considered safer; however, the potential for allergenic reactions cannot be ignored.

Hypertension is a chronic condition and a significant risk factor for cardiovascular and cerebrovascular diseases. Its pathophysiology is intricately associated with the angiotensin-converting enzyme (ACE), which facilitates the conversion of angiotensin I to angiotensin II, a potent vasoconstrictor that plays a crucial role in blood pressure regulation within the renin–angiotensin–aldosterone system. Current therapeutic approaches predominantly utilize synthetic ACE inhibitors, such as captopril and enalapril, which may be associated with adverse effects. Consequently, ACE-inhibitory peptides derived from marine sources have garnered increasing interest as natural alternatives that may aid in blood pressure regulation, particularly within preventive or complementary strategies [6,7,8,9].

There is considerable technological interest in identifying antioxidant ingredients that can serve as alternatives to synthetic antioxidants commonly used in food products, such as Butylated Hydroxyanisole (BHA) and Butylated Hydroxytoluene (BHT). The development of antioxidants capable of exerting in vivo effects is of substantial interest to the nutraceutical industry. The intestinal epithelium is particularly susceptible to redox imbalance and oxidative damage owing to its constant exposure to luminal oxidants, dietary components, xenobiotics, and a dense microbial population. Consequently, dietary strategies aimed at enhancing antioxidant capacity are crucial for mitigating oxidative damage and maintaining mucosal homeostasis [10,11,12]. In this context, peptides derived from marine protein sources have been identified as potent antioxidants suitable for incorporation into food products or direct commercialization as nutraceuticals [13,14,15]. Most cellular screenings have used Caco-2 models, in which fish hydrolysates have been shown to mitigate H_2_O_2_-induced reactive oxygen species (ROS) accumulation and lipid peroxidation. Nevertheless, relatively few studies have examined the antioxidant effects of enteroendocrine models. This gap is significant because enteroendocrine cells secrete various gut hormones, such as glucagon-like peptide-1 (GLP-1), cholecystokinin (CCK), and peptide YY (PYY), which are crucial for regulating digestion, satiety, and metabolic signaling [10]. Furthermore, murine STC-1 cells have been extensively utilized as an in vitro platform for enteroendocrine screening. Excess ROS can promote lipid peroxidation, oxidative modification of proteins and nucleic acids, and mitochondrial dysfunction, collectively affecting enteroendocrine cell physiology and potentially disrupting hormone secretion. Therefore, identifying food-derived compounds (including peptides) capable of reducing intracellular ROS is of interest not only from a barrier protection perspective but also in the context of preserving enteroendocrine signaling [16]. In addition to direct radical scavenging, cellular antioxidant effects may involve the activation of endogenous defenses, which are frequently linked to the Keap1–Nrf2 pathway [17].

Type 2 diabetes is a prevalent chronic disease, particularly in developed countries, and is commonly treated with synthetic drugs that inhibit dipeptidyl peptidase IV (DPP-IV, EC 3.4.14.5), an enzyme involved in incretin inactivation and loss of insulinotropic activity. Consequently, natural peptides with DPP-IV inhibitory activity have gained attention as complementary therapeutic agents [18]. Some authors have reported the ability of protein hydrolysates derived from marine sources to inhibit DPP-IV [19,20,21].

Neurodegenerative and neuropsychiatric disorders also represent major and growing public health challenges. Among the endogenous targets investigated in this context, prolyl endopeptidase (PEP, EC 3.4.21.26), also known as prolyl oligopeptidase (PO), has been associated with neuropathological conditions such as depression, schizophrenia, and Alzheimer’s disease [22]. PEP inhibitors can improve memory by reducing the degradation of endogenous neuropeptides [23]. Although PEP-inhibitory peptides have been found in protein hydrolysates, their in vivo effects remain insufficiently characterized.

Although the above evidence supports the broader relevance of marine bioactive peptides across cardiometabolic and neuro-related targets, translation into practical food and nutraceutical applications requires not only potent activities in vitro but also control over peptide profiles and their stability during gastrointestinal digestion. In this context, tilapia scales remain an underexplored substrate, despite their collagen-rich composition. Previous studies have reported peptides with antioxidant activity (IC_50_ of 0.76 g/L in the ABTS radical scavenging activity assay) and ACE-inhibitory capacity (IC_50_ of 0.73 mg/mL) derived from tilapia scales [3,24]. However, most available studies, while valuable, often overlook two critical aspects of functional food and nutraceutical applications: (i) the comprehensive influence of diverse enzymes on peptide generation and functionality, and (ii) the crucial stability of these peptides after SGID. Therefore, a better understanding of how enzyme selection influences bioactivity and digestive stability (key parameters for industrial implementation) is needed.

The primary aim of this study was to valorize tilapia scales, an underutilized protein source, through enzymatic hydrolysis to produce multifunctional bioactive peptides. The research specifically examined the impact of enzyme selection on peptide functionality, bioactivity, and stability under simulated gastrointestinal conditions by evaluating ACE, DPP-IV, and PEP-inhibitory activities, antioxidant capacity both in vitro and in the enteroendocrine STC-1 cell line, and cell viability. By integrating by-product valorization with functional performance and digestive stability, this study seeks to facilitate the development of nutraceutical ingredients from fish-processing residues.

## 2. Materials and Methods

### 2.1. Chemicals and Reagents

All experimental procedures were conducted utilizing analytical-grade reagents. Bovine hemoglobin, trichloroacetic acid, glycine, ammonium sulfate, bovine serum albumin, aprotinin, vitamin B12, norleucine, and dipeptidyl peptidase IV (DPP-IV) from porcine kidney were procured from Sigma Chemical Co. (St. Louis, MO, USA). The commercial proteases, Esperase 8.0 L and Alkaline Protease, were generously supplied by Novozymes (Bagsværd, Denmark) and BioCat (Troy, VA, USA), respectively. Pepsin, pancreatin, and 2,4,6-Tris(2-pyridyl)-s-triazine (TPTZ) were obtained from Sigma-Aldrich (St. Louis, MO, USA). Angiotensin-converting enzyme (ACE, EC 3.4.15.1) was also sourced from Sigma-Aldrich. Prolyl oligopeptidase (PO, EC 3.4.21.26) from *Flavobacterium meningosepticum* was acquired from the Seikagaku Corporation (Tokyo, Japan). Chromogenic substrates for DPP-IV and PEP (H-Gly-Pro-AMC·HBr and Z-Gly-Pro-AMC, respectively) were purchased from Bachem (Bubendorf, Switzerland).

### 2.2. Sample Collection and Preparation

Tilapia specimens were procured from a local market in Quito, Ecuador, where the fish were purchased already dead for commercial consumption. A total of 10 adult tilapia specimens were used in this study, and the collected scales were pooled prior to processing in order to obtain a representative and homogeneous raw material. The fish scales were manually descaled and thoroughly cleansed with distilled water to eliminate impurities. Subsequently, the scales underwent demineralization as outlined by Mosquera et al. [25]. Prior to demineralization, non-collagenous proteins were extracted, and the scales were subjected to two treatments with a 5% (*w*/*v*) NaCl solution for 30 min, followed by treatment with 0.1 N NaOH for 1 h. Lipid residues were removed using a 10% (*v*/*v*) isobutyl alcohol solution for 30 min. For the demineralization process, the scales were immersed in a 0.5 N EDTA solution overnight. Residual EDTA was eliminated by washing the scales three times with distilled water, followed by treatment with 0.05 M acetic acid for 3 h. Finally, the treated scales were dried in a forced-air oven at 45 °C for 12 h and stored at −20 °C until further analysis.

### 2.3. Determination of Protein Content

The protein content of the scales was determined from the total nitrogen content (AOAC 992.15) [26] using an LECO TruMac Nitrogen analyzer (Leco Corp, AG Geleen, The Netherlands). The protein content was calculated from the total nitrogen using a conversion factor of 5.55. The results are expressed as grams per 100 g of the dried sample.

### 2.4. Amino Acid Analysis

Demineralized scales were dissolved (1 mg/mL) in ultrapure water and further hydrolyzed in sealed vacuum glass at 110 °C for 24 h in the presence of 6 N HCl containing 0.1% phenol and norleucine as an internal standard. After hydrolysis, the samples were dried under vacuum and reconstituted in an application buffer. The hydrolysates were analyzed using an automatic amino acid analyzer (Biochrom 30, Biochrom Ltd., Cambridge, UK) equipped with an LKB Ultropack 8 resin column (Pharmacia LKB Biotechnology Inc., Piscataway, NJ, USA). The results were expressed as grams per 100 g of total amino acids (%).

### 2.5. FTIR-Spectra of the Protein Hydrolysates

Fourier-transform infrared (FTIR) spectra of the hydrolysates were obtained using a PerkinElmer Spectrum 400 FTIR spectrometer (PerkinElmer Inc., Waltham, MA, USA) equipped with an attenuated total reflectance (ATR) accessory. The spectra were recorded over the wavenumber range of 4000–800 cm^−1^, with a spectral resolution of 4 cm^−1^. For each measurement, approximately 1 mg of the dried sample was placed on the ATR crystal and compressed with a flat-tip plunger to ensure optimal contact and the acquisition of well-defined spectral bands. A background spectrum was collected and subtracted using Spectrum software (version 6.3.2; PerkinElmer Inc., Waltham, MA, USA). The spectra are presented as absorbance values at selected wavenumbers within the 4000–800 cm^−1^ range.

### 2.6. Determination of Protease Activity

Protease activity was determined using casein as a substrate according to Lajmi et al. [27]. The enzyme solutions, at different concentrations, were incubated with 1% casein in 50 mM sodium phosphate buffer (pH 8.0) at 38 °C for 12 min. The reaction was stopped with trichloroacetic acid, and the mixture was filtered. The released peptides were quantified using the Folin–Ciocalteu reagent, measuring absorbance at 750 nm. Tyrosine was used as a standard, and one unit of activity was defined as the amount of enzyme releasing 1 μM of tyrosine per minute under the assay conditions.

### 2.7. Protein Hydrolysis and Degree of Hydrolysis (DH)

Enzymatic hydrolysis was conducted utilizing a pH-stat system that maintained a constant pH through the continuous addition of 0.5 M NaOH. Specifically, 2.5 g of the sample (equivalent to 2.43 g of protein) was dispersed in 25 mL of distilled water. The suspension was subsequently adjusted to the optimal pH for each enzyme (pH 9) while the temperature was elevated to the optimal operational value (55 °C). Upon reaching the desired pH and temperature conditions, 110 mg of Alkaline Protease and 500 µL of Esperase were added, corresponding to an enzyme dosage of 20 µUnits/g protein. The pH was consistently maintained throughout the process by the automatic addition of NaOH, ensuring optimal conditions for the enzymatic activity of the protease. The hydrolysis reaction was conducted for a duration of 3 h under controlled conditions. Post-hydrolysis, the enzymes were inactivated by heating the samples at 90 °C for 15 min. The mixtures were then centrifuged at 4200 ×g for 20 min at 4 °C (Beckman Coulter J2-MC, Indianapolis, IN, USA). The supernatants were collected, freeze-dried, and stored at −20 °C until further use.

DH was calculated according to the method described by Alder-Nissen [28]. The total number of peptide bonds (h_tot_) was calculated from the amino acid content analysis data. The results were expressed as the percentage of cleaved peptide bonds (h) relative to the total number of peptide bonds available for proteolysis (Equation (1)):

(1) $$DH\;\left(\%\right)=\frac{h}{h_{total}}x100=\frac{N_{b}\;x\;V_{NaOH}}{MP\;x\;\alpha \;xh_{tot}}x100\;$$

where *V_NaOH_* is the volume of NaOH consumed (mL), *N_b_* is the normality of the base, *MP* is the protein mass (g), *h_tot_* is the total number of peptide bonds in scales (10.70 mEq/g protein) and ∝ is the dissociation degree of the ∝NH_2_ groups released during hydrolysis.

### 2.8. Molecular Weight Profile (MW)

The MW profiles were determined following the methodology of Martínez-Álvarez et al. [29], utilizing a Peptide PC 3.2/30 column (GE Healthcare, Barcelona, Spain). The mobile phase comprised 30% (*v*/*v*) acetonitrile in Milli-Q water (Millipore Co., New Bedford, MA, USA) with 0.1% (*v*/*v*) trifluoroacetic acid (TFA). The flow rate was maintained at 0.1 mL/min. Samples were prepared at a concentration of 10 mg/mL and filtered through 0.45 μm syringe filters prior to injection. Elution was monitored by measuring absorbance at 214 nm. MW calibration was conducted using the following standards: aprotinin (6511 Da), vitamin B12 (1345 Da), hippuryl-histidyl-leucine (429 Da), and glycine (75 Da). The void volume of the column was determined by injecting bovine serum albumin (BSA; 67,000 Da).

### 2.9. Simulated Gastrointestinal Digestion (SGID)

Simulated gastrointestinal digestion was performed according to a previously reported adaptation of the INFOGEST harmonized protocol [30]. Briefly, 2 g of freeze-dried hydrolysate was suspended in 8 mL of simulated salivary fluid (SSF), adjusted to pH 7.0, and incubated under agitation for 2 min at 37 °C. Subsequently, a 4 mL aliquot was collected for further analysis.

The remaining salivary fraction was mixed with 12 mL of simulated gastric fluid (SGF) containing pepsin (6500 U/mL). The pH was adjusted to 3.0, and the mixture was incubated at 37 °C for 2 h under continuous agitation. At the end of the gastric phase, a 4 mL aliquot was collected and heated at 95 °C for 10 min to inactivate pepsin.

For the intestinal phase, 20 mL of simulated intestinal fluid (SIF) containing pancreatin (45 U/mL) was added, and the pH was adjusted to 7.0. The mixture was incubated at 37 °C for 2 h under continuous agitation. Following digestion, samples were heated at 95 °C for 10 min to terminate enzymatic activity and then centrifuged at 13,400 × g for 10 min. The resulting supernatants were collected and stored at −20 °C until further analysis.

The modifications introduced to the original protocol consisted of the collection of aliquots after the oral and gastric phases for intermediate analyses and the thermal inactivation of digestive enzymes prior to storage and bioactivity evaluation. In addition, a digestion blank containing all digestive fluids and enzymes but no protein substrate was prepared and processed under identical conditions.

### 2.10. Biological Activities

#### 2.10.1. Antioxidant Activity

The antioxidant activity of the hydrolysates before and after SGID was evaluated using different methodologies.

##### FRAP Assay

The ferric reducing antioxidant power (FRAP) assay was conducted following the methodology outlined by Calvo et al. (2023) [31]. The FRAP reagent was prepared by combining 25 mL of 0.2 M sodium acetate buffer (pH 3.6), 2.5 mL of 10 mM TPTZ (dissolved in 40 mM HCl), and 2.5 mL of 20 mM FeCl_3_. The undigested samples were prepared in distilled water at a concentration of 10 mg/mL. Subsequently, 10 μL of the sample was mixed with 250 μL of the FRAP reagent and incubated at 37 °C for 30 min. Absorbance was measured at 595 nm using a microplate reader (BMG CLARIOstar Plus; BMG Labtech, Ortenberg, Germany). The calibration curve was established using Mohr salt within the concentration range of 0.1–0.8 mM. Results were expressed as mEq Mohr salt per gram of the dried sample. Each sample was analyzed in triplicate.

##### ABTS Radical Scavenging Activity Assay

The ABTS radical scavenging activity assay was conducted following the methodology outlined by Calvo et al. (2023) [31]. A stock solution of ABTS•^+^ was prepared by mixing 7 mM ABTS with 2.25 mM potassium persulfate (K_2_S_2_O_8_) and allowing the mixture to stand overnight in the dark. The solution was then diluted with distilled water to achieve an absorbance of 0.70 ± 0.02 at a wavelength of 734 nm. In a 96-well microplate, 980 μL of the ABTS working solution was combined with 20 μL of undigested hydrolysate, which was diluted to a concentration of 10 mg/mL in distilled water, and the mixture was incubated for 10 min at 30 °C in the dark. Absorbance was measured at 734 nm using a microplate reader. A calibration curve was constructed using ascorbic acid, and the results were expressed as milligrams of ascorbic acid equivalents per gram of hydrolysate (mg ascorbic acid Equ/g). The assay was performed in triplicate.

##### Ferrous Ion Chelating Activity

The Fe(II)-chelating capacity of the hydrolysates was assessed through the following procedure: the undigested hydrolysates were initially dissolved in distilled water at a concentration of 0.05 mg/mL. Subsequently, 270 μL of this solution was combined with 10 μL of FeCl_2_ (0.72 mM) and 20 μL of 1.8 mM ferrozine. The resulting mixture was incubated for 10 min at room temperature. Absorbance was then measured at 562 nm using a microplate reader. A control sample was prepared by substituting distilled water for the sample. The results were expressed as the percentage of Fe(II)-chelating activity per microgram of the sample:

(2) $$\;\left(\%\frac{Act}{ug}\right)=\frac{\left(\frac{A_{control}-A_{sample}}{A_{control}}\right)\times 100}{S_{a}\;\times V_{used}}\;$$

where A _control_ is the absorbance of the control reaction, A _sample_ is the absorbance of the sample, S_a_ is the sample amount and V _used_ corresponds to the volume (μL) of the solution used.

##### Determination of Intracellular ROS Levels in STC-1 Cells

The intracellular production of reactive oxygen species (ROS) was assessed in murine enteroendocrine STC-1 cells (RRID: CVCL_J405) at passage 17 (ATCC, USA) utilizing an ROS Fluorometric Assay Kit (Biogen Científica, Madrid, Spain), in accordance with the manufacturer’s protocol with minor modifications. The cells were cultured in DMEM supplemented with 10% (*v*/*v*) FBS at 37 °C in a humidified atmosphere containing 5% CO_2_. Upon reaching approximately 70% confluence, the culture medium was removed, and the cell monolayer was rinsed with PBS. Cells were subsequently detached using 0.25% trypsin with 0.02% EDTA and seeded into a black 96-well plate at a density of 10,000 cells per well under standard culture conditions. After 24 h, the medium was replaced with 100 µL of serum-free medium. Subsequently, 10 µL of the sample solution (10 mg/mL in serum-free medium) or serum-free medium (control) was added to each well, and the cells were incubated for 20 h. Following treatment, the supernatant was removed, and the cells were incubated with 100 µL of serum-free medium containing 10 µL of 100 µM 2′,7′-dichlorodihydrofluorescein diacetate (DCFH-DA) for 30 min at 37 °C. The cells were then washed three times with PBS, after which 100 µL of serum-free medium and 10 µL of 500 µM hydrogen peroxide (H_2_O_2_) as an oxidative agent, or ultrapure water (control), were added to each well. The concentration of hydrogen peroxide tested was non-cytotoxic. The plates were incubated for 1 h at 37 °C in the dark. Fluorescence, indicative of intracellular ROS levels, was measured at excitation/emission wavelengths of 485/530 nm using a microplate reader. The results, expressed as the mean of five independent determinations, are reported as fluorescence units.

#### 2.10.2. Cell Viability Assay

Cell viability was determined in murine enteroendocrine STC-1 cells (RRID: CVCL_J405) at passage 14 (ATCC, USA) using the Cell Counting Kit-8 (CCK-8) (Merck). The assay is based on the reduction in the water-soluble tetrazolium salt WST-8 by cellular dehydrogenases.

Cells were cultured in Dulbecco’s Modified Eagle’s medium (DMEM) supplemented with 10% (*v*/*v*) fetal bovine serum (FBS) at 37 °C in a humidified atmosphere containing 5% CO_2_. When the cells reached approximately 70% confluence, the culture medium was removed, and the cell monolayer was rinsed with Ca^2+^/Mg^2+^-free Dulbecco’s phosphate-buffered saline (PBS). The cells were seeded in 96-well plates (10,000 cells/well) and incubated in 100 µL of serum-free culture medium for 24 h to allow cell attachment. Cells were then treated with 10 µL of the digests at different concentrations (2.5, 5 and 10 mg/mL of serum-free medium) for 24 h. Then, 10 µL of CCK-8 reagent was added directly to each well, and the plate was incubated for 2 h at 37 °C. Absorbance was measured at 450 nm using a microplate reader. Cell viability was expressed as a percentage relative to untreated control cells, which were considered to have 100% viability. All experiments were performed at least three times.

#### 2.10.3. Determination of ACE-Inhibitory Activity

The potential antihypertensive activity of the hydrolysates before and after SGID was evaluated by measuring the inhibition of angiotensin-converting enzyme (ACE, EC 3.4.15.1), following the method described by Sentandreu and Toldrá [32], with slight modifications. Assays were performed in 384-well microplates with a final reaction volume of 100 µL. For the sample wells, 20 μL of ACE solution (dilution 1/50, *v*/*v*), 10 μL of hydrolysate at different concentrations, and 40 μL of Tris–HCl buffer (150 mM Tris–HCl containing 1.125 M NaCl, pH 8.3) were added. For the control wells, 20 μL of ACE solution and 70 μL of Tris–HCl buffer were used. The mixtures were pre-incubated at 37 °C for 15 min. The reaction was initiated by adding 30 μL of the fluorogenic substrate Abz-Gly-Phe(NO_2_)-Pro (0.9 mM) to the sample and control wells using the injector system of the microplate reader. Fluorescence was monitored for 15 min at λexc 320 nm and λem 420 nm, with measurements recorded every minute. The maximum linear increase in fluorescence per minute was calculated for each sample. The ACE-inhibitory activity of the hydrolysates was tested at different concentrations and expressed as IC_50_, defined as the concentration of hydrolysate required to inhibit 50% of ACE activity. There were three replicates per sample.

#### 2.10.4. Determination of DPP-IV Inhibitory Activity

The hypoglycemic potential of the hydrolysates was evaluated before and after SGID by measuring the inhibition of dipeptidyl peptidase IV (DPP-IV, EC 3.4.14.5) following the method described by Sila et al. [23], with slight modifications. Assays were performed in 96-well microplates with a final reaction volume of 300 μL each. For the sample wells, 20 μL of DPP-IV solution, 30 μL of hydrolysate (at different concentrations), and 150 μL of 0.1 M Tris–HCl buffer (pH 8.0) were added. For the control wells, 20 μL of DPP-IV solution and 180 μL of Tris–HCl buffer were used. The mixtures were pre-incubated at 37 °C for 15 min. The reaction was initiated by adding 100 μL of the fluorogenic substrate H-Gly-Pro-AMC·HBr (0.01 mM in the working buffer) to both the sample and control wells using the injector system of the microplate reader. Fluorescence was monitored for 15 min at λexc 340 nm and λem 440 nm, with measurements recorded every minute at these wavelengths. The maximum linear increase in fluorescence per minute was calculated for each well. The DPP-IV-inhibitory activity of the hydrolysates was expressed as IC_50_, defined as the concentration of hydrolysate required to inhibit 50% of DPP-IV activity. There were three replicates per sample.

#### 2.10.5. Determination of PEP-Inhibitory Activity

PEP-inhibitory activity was determined before and after SGID following the same procedure described for DPP-IV inhibition, with slight modifications, according to Sila et al. [23]. Briefly, assays were performed in 96-well microplates using 0.1 M sodium phosphate buffer (pH 7.0) as the working solution. For the sample wells, 20 μL of PEP solution, 30 μL of hydrolysate (at different concentrations), and 150 μL of 0.1 M working buffer were added. For the control wells, 20 μL of PEP and 180 μL of working buffer were used. The mixtures were pre-incubated at 37 °C for 15 min. The reaction was initiated by adding 100 μL of Z-Gly-Pro-AMC (0.025 mM in the working buffer). Fluorescence was monitored under the same conditions as described for DPP-IV inhibitory activity. The maximum linear increase in fluorescence per minute was calculated for each well. The PEP-inhibitory activity was expressed as IC_50_, defined as the concentration of hydrolysate required to inhibit 50% of PEP activity. There were three replicates per sample.

### 2.11. Statistical Analysis

Statistical analyses were performed using Statgraphics Centurion XVI software (StatPoint, Inc. Warrenton, VA, USA). Differences between treatments were assessed using one-way analysis of variance (ANOVA), followed by a least significant difference (LSD) test at a significance level of *p* < 0.05. An independent samples Student’s *t*-test was performed for pairwise comparison. All experiments were performed at least in triplicate.

## 3. Results and Discussion

### 3.1. Amino Acid Analysis

Table 1 shows the amino acid profile of tilapia scale protein. Glycine was the predominant amino acid, accounting for 19.70%, followed by proline (12.12%), hydroxyproline (11.98%), and glutamic acid/glutamine (9.73%).

The amino acid profile is characteristic of collagen-rich matrices and is consistent with previous reports on red tilapia (*Oreochromis* sp.) [3] and Nile tilapia (*Oreochromis niloticus*) scales [33]. However, compared with these studies, a lower proportion of alanine was observed in the present study, which is a trend reported by Zhang et al. [24]. Similarly, Mohammad et al. [34] identified glycine as the most abundant amino acid in gelatin derived from tilapia scales, followed by proline, with values comparable to those obtained in this study.

The presence of hydrophobic amino acids is associated with enhanced radical scavenging activity, as these residues improve peptide solubility in non-polar environments [35]. Additionally, low concentrations of cysteine and isoleucine were detected, consistent with the typical composition of type I collagen found in fish scales. Notably, the high proportion of hydrophobic amino acids, particularly glycine, proline, alanine, valine, leucine, and phenylalanine, may promote interactions between peptides and the hydrophobic regions of enzyme active sites, thereby contributing to ACE-, DPP-IV-, and PEP-inhibitory activities. In particular, proline-containing peptides have frequently been associated with ACE and DPP-IV inhibition due to the affinity of these enzymes for substrates containing proline residues. Likewise, PEP-inhibitory activity has also been related to peptides rich in proline because PEP preferentially hydrolyzes peptide bonds adjacent to proline residues. Furthermore, amino acids such as tyrosine, cysteine, methionine, and histidine may contribute to antioxidant activity through radical scavenging, electron donation, and metal-chelating mechanisms. The abundance of glycine and proline may also favor peptide interactions with the lipid bilayer of cell membranes, facilitating peptide permeability, stability, and biological functionality [3].

### 3.2. Degree of Hydrolysis (DH)

The highest degree of hydrolysis (DH) was achieved with Alkaline Protease, reaching 12.4%, a value comparable to that reported by Sierra-Lopera et al. [3], who observed DH values ranging from 16.7% to 17.6%. In contrast, Esperase exhibited the lowest DH at 6.2%, an intermediate value that reflects significant differences in the catalytic efficiency of the enzymes evaluated. These variations can be attributed to both enzyme specificity and substrate characteristics, as protein composition and amino acid profiles directly influence susceptibility to hydrolysis. In this context, proteolytic efficiency depends on the ability of each enzyme to recognize and cleave specific peptide bonds within a protein sequence [36].

Zhang et al. [24] reported DH values of 7.8% using Alcalase after 180 min of hydrolysis; however, the application of a prior hydrothermal pretreatment increased the DH to 12.9%, highlighting substrate accessibility as a key factor governing hydrolysis efficiency. Similarly, previous studies on the hydrolysis of fish scale gelatin have employed enzymes such as trypsin, papain, neutral protease, and Alkaline Protease, with the latter achieving the highest DH values of up to 48.1% [37]. However, these results are not directly comparable, as these studies used pre-extracted gelatin, a partially denatured and more accessible form of collagen. In contrast, the present study employed demineralized tilapia scales, in which collagen presumably retains its highly organized structure. This highly ordered structure, characterized by crystalline regions and cross-links, restricts access to peptide bonds and influences enzyme–substrate interactions, thereby negatively affecting the catalytic efficiency of proteases [38]. In this context, the observed differences in DH among the evaluated serine proteases can be attributed to variations in enzymatic specificity, conformational flexibility of the active site, and stability under reaction conditions. Alkaline Protease exhibited the highest hydrolytic capacity, indicating lower substrate specificity and greater structural adaptability at its binding sites (S1–S3) [39]. This allows it to accommodate structurally restrictive residues, such as proline, which is a predominant feature of collagen. This lower steric restriction favors an increase in the frequency of catalytic events and, consequently, a higher rate of peptide bond cleavage [39]. In contrast, Esperase showed lower DH, which can be explained by its greater specificity towards hydrophobic residues at defined positions, thereby limiting its action on repetitive sequences rich in glycine and proline. In addition, the presence of proline near the cleavage site introduces conformational constraints that reduce the catalytic efficiency of subtilisin-like proteases [40]. The heterogeneous and partially mineralized nature of the scales must also be considered, as it can restrict enzymatic accessibility. This favors the performance of enzymes with lower specificity and greater surface interaction capacity [41].

### 3.3. MW Profile Before, During and After SGID

**Figure 1:**
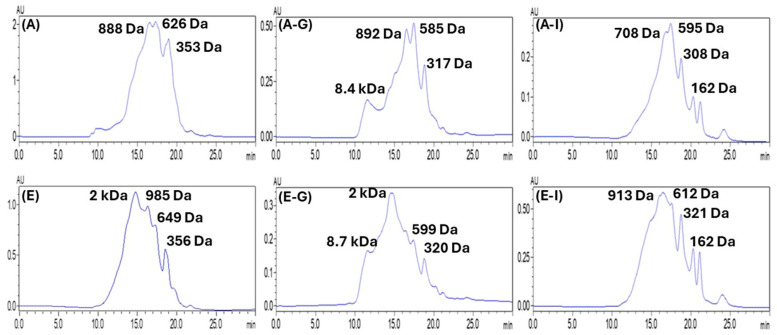
MW profiles of AP- (**A**) and E-hydrolysates (**E**) before, during, and after SGID. (**A-G**) AP-hydrolysate, gastric phase; (**A-I**) AP-hydrolysate, intestinal phase; (**E-G**) E-hydrolysate, gastric phase; (**E-I**) E-hydrolysate, intestinal phase.

Size-exclusion chromatography revealed notable differences in the MW distribution of the hydrolysates obtained with Alkaline Protease (AP-hydrolysate) and Esperase (E-hydrolysate) before and after SGID (digests), as shown in Figure 1.

The AP-hydrolysate was dominated by low-MW peptides, as evidenced by a major peak at 888 Da, corresponding to peptides of approximately 5–7 residues (Figure 1A). This suggests extensive fragmentation of the collagenous scale matrix, leading to the formation of short-peptide species. Following gastric digestion, high-MW polypeptides (8.4 kDa) emerged, which were absent from the MW profile of the undigested hydrolysate (Figure 1). This observation suggests that aggregated or poorly soluble material present in the initial hydrolysate (likely removed during sample centrifugation and filtration prior to injection) became soluble and partially hydrolyzed under gastric conditions. Meanwhile, the peptide population around 890 Da remained stable, and additional low-MW fractions appeared, including peaks at 585 Da (3–4 residues) and 317 Da (likely dipeptides). After intestinal digestion (Figure 1A-I), the 8.4 kDa fraction disappeared, and the MW profile shifted towards a predominance of peptides below 800 Da, with notable populations at 708, 595, and 308 Da. The disappearance of the 8.4 kDa fraction indicates further enzymatic breakdown into smaller peptides. In contrast, the E-hydrolysate exhibited an MW distribution characterized by a predominance of higher-MW peptides (2 kDa and 985 Da), alongside a substantial proportion of lower-MW peptides (649 and 356 Da) (Figure 1E). This profile suggests less extensive initial fragmentation compared to the AP-hydrolysate, despite the presence of short peptides. Following simulated gastric digestion, the E-hydrolysate showed an increase in higher-MW components (Figure 1E-G), as observed with the AP-hydrolysate, with a dominant population around 2 kDa and peptides below 600 Da. After simulated intestinal digestion (Figure 1E-I), the MW distribution shifted further towards peptides below 920 Da, with a marked increase in peptides around 320 Da, reflecting the progressive breakdown of peptides into shorter forms during SGID.

Despite their differing initial profiles, both hydrolysates converged after SGID towards mixtures enriched in very low-MW peptides (<600 Da; mainly di- and tripeptides), while larger fragments were transiently generated (mainly after the gastric step) or persisted at low abundance, suggesting a heterogeneous digestion pattern. A similar coexistence of high- and low-MW fractions has been reported for tilapia scale hydrolysates obtained using other proteases (e.g., Alcalase and 73,071 protease), where predominant higher-MW fractions were observed alongside detectable <400 Da peptides, highlighting the role of enzyme specificity and substrate structure in governing peptide release [42]. Mechanistically, these trends are consistent with the collagen-rich nature of fish scales: type I collagen contains a highly stable triple helix dominated by the Gly–Pro–X motif, which confers resistance to proteolysis [43]. Broad-spectrum alkaline proteases may therefore yield more extensive fragmentation than more specificity-driven proteases, contributing to the stronger low-MW- shift observed for AP-hydrolysate. Conversely, Esperase-type proteases preferentially cleave peptide bonds adjacent to hydrophobic residues, and the limited accessibility and/or frequency of such residues within collagen repetitive regions may restrict cleavage site availability, resulting in a predominance of higher-MW peptides while still allowing the formation of smaller fractions [44,45]. Finally, although peptides longer than approximately five residues are generally expected to be susceptible to gastrointestinal degradation, the persistence of some fragments suggests sequence-dependent stability. In particular, collagen-derived peptides containing proline or hydroxyproline at the C-terminus can display enhanced resistance to digestive enzymes, which may contribute to the enrichment of collagen di- and tripeptides after SGID [46].

### 3.4. Fourier-Transform Infrared Spectroscopy (FTIR) Spectra of the AP- and E-Hydrolysates

**Figure 2:**
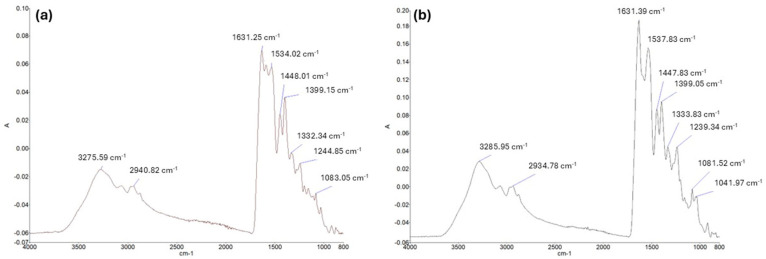
FTIR spectra of AP-hydrolysate (**a**) and E-hydrolysate (**b**).

The FTIR spectra of the hydrolysates are shown in Figure 2. Both samples exhibited characteristic absorption bands of proteins and peptides, including Amide A, Amide I, Amide II, and Amide III bands, confirming the proteinaceous nature of the hydrolysates [47,48].

In both spectra, a broad band was observed in the 3700–3000 cm^−1^ region, peaking within the Amide A range (3275–3285 cm^−1^). The Amide A band primarily arises from N–H stretching vibrations of peptide bonds and is highly sensitive to hydrogen-bond strength. In dried hydrolysates, it often overlaps with the broad O–H stretching envelope of adsorbed water. The band in the 3700–3000 cm^−1^ region appeared slightly sharper for the E-hydrolysate, possibly indicating differences in hydration and hydrogen-bonding environments, given that Amide A (mainly N–H stretching) is highly sensitive to hydrogen bonding and typically overlaps with the broad O–H stretching contribution from residual/adsorbed water in dried hydrolysates.

The Amide I band (1630–1635 cm^−1^) was the most intense feature in both spectra, mainly linked to the C=O stretching vibration of the peptide bond, which suggests a peptide-rich composition in both hydrolysates. Differences were observed in the 1580–1600 cm^−1^ region, with the AP-hydrolysate showing greater intensity. This region includes contributions from the carboxylate (COO^−^) stretching vibrations of acidic amino acid side chains, such as those of aspartic and glutamic acids, along with signals from exposed aromatic residues after hydrolysis, indicating compositional differences between the two hydrolysates [48,49]. The band corresponding to the E-hydrolysate also appeared slightly sharper, potentially due to differences in hydrogen-bonding environments or a higher relative exposure of free amino groups, consistent with the presence of low-MW peptides observed in the corresponding chromatographic profile (Figure 1).

The Amide II band (1534–1537 cm^−1^), characterized by N–H bending coupled with C–N stretching vibrations, exhibited minor variations among the samples. These variations may suggest differences in peptide conformation attributable to enzymatic treatment. No significant differences were observed in the CH_2_ and CH_3_ deformation bands at 1448 and 1399 cm^−1^, respectively, indicating comparable contributions from the aliphatic amino acid side chains [49]. Similarly, no notable changes were detected in the Amide III region (1300–1200 cm^−1^), which is typically associated with C–N and N–H vibrations in collagen-derived matrices [50].

Variations were detected in the 1200–1100 cm^−1^ region, typically associated with C–O and C–O–C stretching vibrations within the carbohydrate “fingerprint” region. These spectral bands were more pronounced in the AP-hydrolysate, indicating a greater relative contribution of sugar chains, possibly associated with peptides or free amino acids [51]. Finally, the pronounced bands observed at approximately 1100–1030 cm^−1^ likely indicate the presence of residual mineral components within the hydrolysates [50].

### 3.5. Biological Activities of the Protein Hydrolysates

#### 3.5.1. Effect of the Digested Hydrolysates on STC-1 Cells Viability

**Figure 3:**
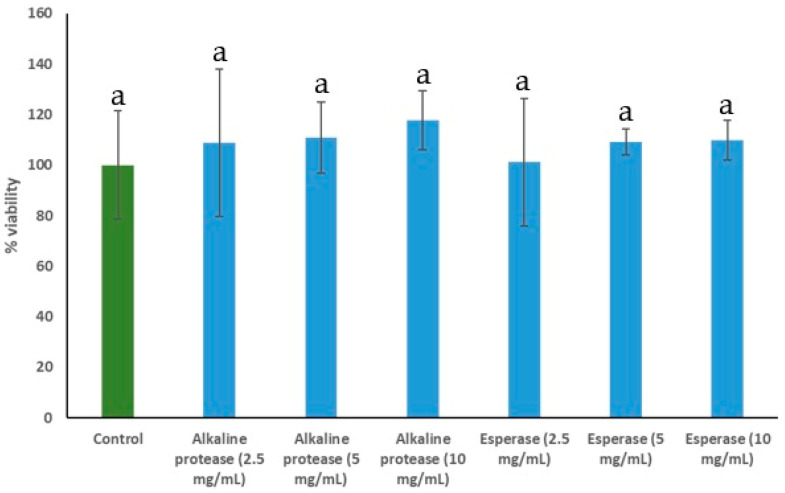
Viability of STC-1 cells after 24 h of exposure to digests (2.5–10 mg/mL) and assessed using the Cell Counting Kit-8 (CCK-8). Data are expressed as a percentage relative to the untreated control (100%) and are presented as the mean ± SD. Different letters indicate significant differences among treatments according to one-way analysis of variance (ANOVA) followed by Fisher’s LSD post hoc test (p < 0.05), whereas identical letters indicate no statistically significant differences.

The viability of STC-1 enteroendocrine cells in contact with the digests was evaluated at concentrations ranging from 2.5 to 10 mg/mL (Figure 3).

No significant changes in STC-1 cell viability were observed for any of the digests compared to the control (*p* > 0.05), indicating that the samples did not exert cytotoxic effects under the tested experimental conditions. A slight upward trend in viability was observed with increasing concentration, particularly for the digested AP-hydrolysate, although these differences were not statistically significant. Overall, these results suggest that the digests are non-cytotoxic to STC-1 cells up to 10 mg/mL after 24 h of exposure. It should be noted that the CCK-8 assay primarily reflects cellular metabolic activity (via dehydrogenase activity) rather than directly measuring cell number or proliferation. Therefore, changes in metabolic activity may not strictly correlate with cell viability and could potentially mask subtle cytotoxic or proliferative effects. Complementary endpoints, such as LDH release, apoptosis/necrosis markers, or, in differentiated intestinal models, barrier integrity assays, would further strengthen safety assessments.

#### 3.5.2. Antioxidant Activities of Protein Hydrolysates Before and After SGID

The antioxidant capacity of non-digested tilapia scale hydrolysates was evaluated using complementary in vitro assays (ABTS radical scavenging activity assay, ferric reducing antioxidant power (FRAP), and Fe(II)-chelating activity) (Table 2).

The ABTS assay evaluates antioxidant capacity by assessing the ability of the sample to neutralize the ABTS•^+^ radical through electron transfer and/or hydrogen atom donation. As shown in Table 2, both hydrolysates exhibited nearly identical ABTS values, suggesting that, under the tested conditions, neither the degree of hydrolysis (DH) nor the primary differences in peptide size distribution resulted in a discernible difference in ABTS-scavenging performance. Evidence regarding ABTS activity specifically for fish scale-derived hydrolysates remains scarce. Nonetheless, reviews of marine by-product hydrolysates (including scales) consistently indicate that these matrices can produce peptide mixtures with significant antioxidant potential across chemical assays such as ABTS [13]. Although many studies focus on muscle or whole-tissue hydrolysates, the general principles are applicable to scale hydrolysates because antioxidant outcomes are largely determined by the peptide pool generated (sequence, composition, and size) rather than solely by tissue origin. In a scale-specific instance, Chen et al. [52] reported an ABTS scavenging activity of 16.41% for papain hydrolysates derived from grass carp scale gelatin. While the present study expressed antioxidant activity using different units (18.76 mg ascorbic acid Equ/g), the results demonstrate similar antioxidant potential for fish scale-derived hydrolysates, even at lower concentrations.

In contrast, differences were observed in the FRAP and metal-binding capacities of the hydrolysates. The AP-hydrolysate exhibited markedly higher Fe(II)-chelating activity than the E-hydrolysate (Table 2). This result indicates a greater Fe(II)-chelating capacity in the AP-hydrolysate, which may be associated with differences in the peptide populations generated by the two enzymes. However, the specific peptides responsible for this activity were not identified in the present study. Comparable chelating activities have been reported for other marine hydrolysates, although typically at substantially higher concentrations. For example, shrimp waste hydrolysates achieved 85.3–98% chelation at 1–3 mg/mL, whereas goby muscle hydrolysates reached 90–97% chelation at 5 mg/mL [53,54]. This chelating capacity may be associated with the presence of histidine residues (as suggested by the amino acid profile) and contributions from acidic and basic residues that facilitate coordination with metal ions [53,54]. Furthermore, the AP-hydrolysate showed a significantly (Student’s *t*-test) higher FRAP value than the E-hydrolysate (Table 2), which would be ascribed to differences in peptide composition, size, and sequence, and not to the enzyme employed [55]. Antioxidant peptides are often associated with low-MW fractions (commonly <1 kDa), and the AP-hydrolysate was enriched in these peptides, which could be the main contributors to the antioxidant activity. However, peptides within the 3–10 kDa range have also been reported to exhibit antioxidant activity [56], suggesting that antioxidant effects cannot be attributed exclusively to very small peptides. In this context, peptide fractionation strategies, such as ultrafiltration or chromatographic separation, followed by the evaluation of antioxidant activity in individual fractions, would help to establish a stronger relationship between peptide MW distribution and the observed antioxidant properties. The E-hydrolysate contained a larger proportion of peptides of approximately 2 kDa that could, therefore, exert an antioxidant effect. Moreover, the presence of specific residues, particularly glycine, proline, and hydrophobic residues (alanine, valine, leucine, isoleucine, and methionine), has been linked to radical scavenging and peptide stability [3,57], and their presence may contribute to the observed antioxidant effects of the peptides.

**Figure 4:**
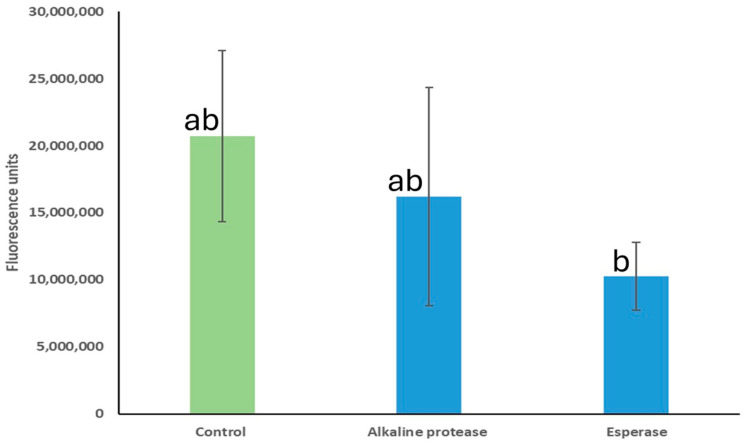
Antioxidant effects of digests in H_2_O_2_-stimulated STC-1 cells (ROS assay). A lower fluorescence intensity indicates greater inhibition of hydrogen peroxide-induced oxidation. The results are expressed as mean ± SD. Different letters indicate statistically significant differences among samples according to one-way ANOVA followed by Fisher’s LSD post hoc test (*p* < 0.05).

The antioxidant capacity of the digests was not assessed using ABTS, FRAP, or Fe(II)-chelating activity assays. Instead, antioxidant activity was evaluated in the enteroendocrine cell line STC-1 using an ROS assay (Figure 4). Therefore, the results obtained for the digests cannot be directly compared with the chemical antioxidant assays performed on the undigested hydrolysates, as these approaches evaluate different biological endpoints. While ABTS, FRAP, and metal-chelating assays measure chemical antioxidant capacity in cell-free systems, the ROS assay evaluates the ability of the digests to modulate oxidative stress under cellular conditions (Figure 4).

Both digests exhibited antioxidant effects in the STC-1 model; however, only the intestinal digest obtained from the Esperase hydrolysate produced a statistically significant reduction in intracellular ROS levels (*p* < 0.05) under the experimental conditions tested, corresponding to approximately 50% inhibition relative to the H_2_O_2_-stimulated control. Similar antioxidant effects of marine-derived protein hydrolysates have previously been reported in intestinal cell models [58,59]. The observed reduction in ROS levels indicates that compounds present in the digest were able to attenuate oxidative stress under the experimental conditions employed. Although several mechanisms, including radical scavenging, metal chelation, inhibition of lipid peroxidation, and modulation of endogenous antioxidant defense systems, have been proposed for food-derived peptides in previous studies, the specific compounds and mechanisms responsible for the effects observed in the present study were not investigated. Likewise, although activation of pathways such as Nrf2/ARE has been reported for some bioactive peptides in intestinal cell models [60,61,62,63], no mechanistic analyses were performed in this work, and therefore the involvement of these pathways remains speculative. Although this signaling axis was not evaluated in the present study, it is plausible that the peptides present in the digests could engage in similar mechanisms in enteroendocrine cells. Finally, peptide sequence and physicochemical properties appear to influence bioactivity. Espíndola et al. [60] conducted a study on digestion-resistant whey peptides generated by SGID. They reported that the resistant peptide fraction was enriched in hydrophobic and acidic amino acids while showing average surface hydrophobicity and also attenuated H_2_O_2_-induced ROS production in Caco-2 cells. This observation supports the idea that specific peptide features (including size and hydrophobicity) may contribute to cellular antioxidant effects.

#### 3.5.3. ACE-Inhibitory Activity Before and After SGID

The inhibitory activities of ACE, DPP-IV, and PEP were assessed in both the undigested hydrolysates (Table 2) and following gastric and intestinal digestion (Table 3).

Both undigested hydrolysates exhibited significant ACE-inhibitory activity, with no statistically significant differences observed in their IC_50_ values, which were approximately 14 µg/mL (Table 2). These low IC_50_ values indicate a high inhibitory potency and fall within the same order of magnitude as reference inhibitors such as enalapril (3.62 µg/mL), with the advantage that pharmacological ACE inhibitors can be associated with adverse effects [64]. The relatively minor difference observed between the AP- and E-hydrolysates at baseline indicates that the hydrolysis of this collagen-rich matrix consistently produces peptide populations with significant affinities for ACE, largely irrespective of the enzyme employed at this stage. The IC_50_ values obtained in the present study are within the lower range of those reported for fish by-product protein hydrolysates in the available literature. While other researchers have reported the ACE-inhibitory effects of protein hydrolysates from fish scales, the effectiveness observed was significantly lower than that presented in this study. Akagündüz et al. [64], obtained a protein hydrolysate of sea bream scales and calculated a low IC_50_ value for a purified fraction (59.0 µg/mL). Zhang et al. [24] reported an IC_50_ value of 0.79 mg/mL for a hydrolysate of tilapia scales gelatin obtained using Alcalase. Da Silva Bernardo et al. [65] reported an ACE-inhibitory activity of 71.2% in tilapia scale hydrolysates at a concentration of 3.3 mg/mL, while Fahmi et al. [66] identified ACE-inhibitory peptides from sea bream scales with an IC_50_ of 0.57 mg/mL. Moreover, the ability of protein hydrolysates obtained from sea bream scales to decrease blood pressure in spontaneously hypertensive rats has been documented [67].

Several studies have reported ACE-inhibitory activity in peptides derived from fish and fish by-products. For example, hydrolysates obtained from grass carp showed an IC_50_ value of 0.872 mg/mL, while Atlantic salmon (*Salmo salar*) hydrolysates presented an IC_50_ of 7.91 mg/mL. Similarly, Alaska pollock (*Theragra chalcogramma*) hydrolysates exhibited an IC_50_ value of 3.41 mg/mL, whereas peptides derived from black rockfish (*Sebastes schlegelii*) showed stronger inhibitory activity with an IC_50_ of 0.62 mg/mL [7,8]. In comparison, the hydrolysates evaluated in the present study exhibited markedly lower IC_50_ values, suggesting a high ACE-inhibitory potential under the in vitro conditions evaluated. These differences may be associated with variations in protein source, enzymatic hydrolysis conditions, peptide composition, and molecular weight distribution.

SGID was associated with a marked increase in the measured ACE-inhibitory activity of both hydrolysates (Table 3). During the gastric phase, the IC_50_ decreased to around 5–6 µg/mL. Following the intestinal phase, ACE inhibition remained more or less constant or even increased slowly, reaching approximately 4.6–6.7 µg/mL, with the E-hydrolysate being the most potent inhibitor after the intestinal phase. These results suggest that gastrointestinal digestion modified the peptide profile and was associated with an increase in ACE-inhibitory potency. However, because digestion blanks were not evaluated, a contribution from peptides originating from the digestive enzymes cannot be completely excluded. These results are consistent with the possibility that sequential pepsin–pancreatin digestion generated peptides with enhanced ACE-inhibitory activity. Nevertheless, the specific origin of the inhibitory peptides could not be established in the present study [68]. In addition, the increase in potency observed after SGID is compatible with the idea that shorter peptides often interact more efficiently with the ACE active site and subsites, improving their inhibitory efficacy and potential bioavailability [69].

When benchmarked against published SGID data from marine sources, the ACE-inhibitory potency achieved in this study was remarkably high. For example, salmon gelatin hydrolysates after SGID have been reported with IC_50_ values in the 0.19–0.14 mg/mL range across gastric and intestinal phases [70], tuna hydrolysate digests ranging from 0.24 to 1.15 mg/mL [46], and digested shrimp hydrolysates around 220 µg/mL [71]. These values are markedly higher (i.e., less potent) than those obtained in the present study (µg/mL range). Finally, the post-intestinal IC_50_ values for both hydrolysates are comparable to the values reported for reference inhibitors such as enalapril (IC_50_ = 3.62 µg/mL) and a synthetic peptide previously reported with an IC_50_ value of 6.6 µg/mL [72]. Nevertheless, these hydrolysates are not intended to replace pharmacological therapy; rather, they support the potential of tilapia scales as a promising source of peptides associated with ACE-inhibitory activity following simulated gastrointestinal digestion, although the specific contribution of digestion-derived peptides from the substrate and digestive enzymes remains to be clarified.

Previous studies have reported that aromatic and branched-chain amino acids, including isoleucine, valine, phenylalanine, and tyrosine, are frequently found in peptides exhibiting ACE-inhibitory activity. Likewise, several ACE-inhibitory peptides with low molecular weight, including proline-containing dipeptides, have been identified from different protein sources [73]. Given the collagenous nature of tilapia scales and their relatively high content of glycine, proline, and hydroxyproline, the raw material may be suitable for the generation of peptides with ACE-inhibitory potential. However, the peptides responsible for the activity observed in the present study were not identified. Therefore, any association between the observed ACE-inhibitory activity and specific peptide sequences, amino acid residues, or structure–activity relationships should be considered hypothetical and based on previous literature. Confirmation of the peptides responsible for the observed activity will require LC-MS/MS peptide identification and further mechanistic studies.

#### 3.5.4. DPP-IV Inhibitory Activity Before and After SGID

The hydrolysates derived from tilapia scales demonstrated significant DPP-IV inhibitory activity, as indicated in Table 2. Both hydrolysates exhibited comparable and substantial inhibitory efficacy, with IC_50_ values approximately ranging from 0.65 to 0.70 mg/mL. This observation stands in contrast to the pattern identified for ACE inhibition, implying that both enzymes enhance the release of peptides more closely associated with ACE inhibition than with DPP-IV. A direct correlation between the DH and DPP-IV inhibitory activity has been documented in certain systems, which may account for these observed differences [74], although the final outcome remains dependent on the peptide motifs generated. From a structural perspective, both hydrolysates contained a high proportion of peptides below 1000 Da. However, the E-hydrolysate also presented a broader peptide size distribution, including a prominent fraction of approximately 2 kDa. Although larger peptides are generally associated with lower inhibitory efficiency owing to reduced accessibility to the DPP-IV active site [75], the overall inhibitory response is likely driven by the presence and abundance of specific sequence motifs rather than size alone. In this regard, DPP-IV preferentially cleaves X–Pro and X–Ala dipeptides from substrates composed of three or more amino acids, and such proteolytic susceptibility can compromise the integrity of inhibitory peptides, potentially lowering their apparent activity depending on the peptide pool generated [76]. Consistent with this, peptides containing proline have frequently been associated with strong DPP-IV inhibitory activity [77]. Given the relatively high proline content of tilapia scales, the raw material may be suitable for generating peptides with DPP IV inhibitory potential. However, no peptide sequencing was performed in the present study, and therefore the peptides responsible for the observed activity remain unidentified. Compared with the literature, the DPP-IV inhibitory potency observed here is within the range reported for marine-derived peptides. Hydrolysates produced by *Bacillus* sp. from a mixture of fish scales and ground feathers have been reported to inhibit DPP-IV activity by 65–73%. However, the present results indicate higher inhibitory potency despite using a non-enriched substrate consisting solely of tilapia scales [65]. More broadly, marine-derived DPP-IV inhibitory peptides have been reported with IC_50_ values spanning approximately 0.14 to 5 mg/mL [70]. Thus, the values obtained in this study fall within this range and are among the lower reported values, supporting a relevant inhibitory potential.

After SGID, DPP-IV inhibition showed markedly different behavior compared to ACE. Following gastric and intestinal digestion, DPP-IV inhibition was detectable, but with reduced potency relative to that of the undigested hydrolysates (Table 2 and Table 3). Specifically, the IC_50_ increased to around 1.1 mg/mL (gastric phase) and 2.1 mg/mL (intestinal phase) for the AP-hydrolysate, and to 1.6 mg/mL (gastric phase) and 1.1 mg/mL (intestinal phase) for the E-hydrolysate (Table 3), indicating a loss of activity in both digests when compared with the undigested hydrolysates. This reduction is consistent with the action of gastrointestinal proteases, which can degrade or transform initially active peptides, leading to the depletion of inhibitory sequences and/or the formation of fragments with lower affinity for the DPP-IV active site. However, the specific peptide sequences affected during digestion could not be determined because peptide identification was beyond the scope of the present study. Although intestinal digestion improved the DPP-IV inhibitory activity of the E-hydrolysate, the overall results suggest that simulated gastrointestinal digestion may compromise the stability of some DPP-IV inhibitory peptides derived from tilapia scales. Therefore, future studies should focus on strategies to enhance peptide stability during digestion, such as encapsulation or other protective delivery systems, in order to preserve their bioactivity and improve their bioavailability [35].

#### 3.5.5. Evaluation of PEP-Inhibitory Activity Before and After SGID

Both hydrolysates exhibited similar PEP-inhibitory activity (IC_50_ = 0.63–0.72 mg/mL), with the E-hydrolysate showing the lowest IC_50_ value (Table 2), although no significant differences were observed between samples. These results demonstrate that tilapia scale hydrolysates possess relevant PEP-inhibitory activity and support the potential of this by-product as a source of bioactive peptides. The observed inhibitory activity was comparable to that reported for other marine-derived hydrolysates. For example, Sila et al. [23] reported PEP-inhibitory activity in barbel (*Barbus callensis*) gelatin hydrolysates, with IC_50_ values ranging from 0.91 to 3.79 mg/mL depending on the protease employed. Collectively, these comparisons indicate that tilapia scale hydrolysates exhibit competitive PEP-inhibitory activity and highlight the underexplored potential of this fish by-product.

Although the E-hydrolysate exhibited slightly greater inhibitory activity, the peptides responsible for this effect were not identified. Therefore, any relationship between the observed activity and the peptide profiles generated by the different enzymes remains speculative. Previous studies have suggested that proline-containing peptides may contribute to PEP inhibition [35], and Esperase has been reported to generate peptide profiles enriched in proline-containing sequences [23]. However, peptide identification was not performed in the present study, and therefore it cannot be determined whether similar peptides were generated in the hydrolysates evaluated here. Importantly, no clear relationship between DH and PEP-inhibitory activity was observed. Although previous studies have highlighted the importance of peptide sequence and structural characteristics in PEP inhibition [78], the absence of peptide identification prevents the establishment of direct structure–activity relationships. Similar observations have been reported for marine protein hydrolysates from cod muscle proteins [79], sardine and tuna by-products [46], and fish collagen sources [27]. Overall, the limited number of studies specifically addressing PEP-inhibitory activity in fish scale hydrolysates underscores the novelty and relevance of the present findings.

The stability of PEP-inhibitory activity was also evaluated following SGID. After the gastric phase, IC_50_ values remained comparable to those of the undigested hydrolysates (Table 3), indicating that gastric digestion did not substantially modify the measured PEP-inhibitory activity. Conversely, a marked reduction in activity was observed after the intestinal phase. For the AP-hydrolysate, the IC_50_ exceeded 2.5 mg/mL, whereas the E-hydrolysate exhibited an IC_50_ of 1.89 mg/mL. These results indicate that gastrointestinal digestion altered the peptide composition of the hydrolysates and was associated with reduced PEP-inhibitory activity. However, because peptide identification was not performed, the specific changes responsible for this reduction could not be determined. Consequently, further studies will be required to identify the peptides associated with PEP inhibition and to evaluate strategies for preserving this activity during gastrointestinal digestion.

## 4. Conclusions

Overall, this study demonstrated that hydrolysates obtained from tilapia scales exhibited ACE-inhibitory, DPP-IV-inhibitory, and PEP-inhibitory activities, as well as antioxidant activity under the in vitro conditions evaluated. Simulated gastrointestinal digestion was associated with increased ACE-inhibitory activity, while the digests showed no cytotoxic effects, and the intestinal digest of the Esperase hydrolysate reduced intracellular ROS levels in STC-1 cells. These findings highlight the potential of tilapia scales as a source of bioactive peptides and support the valorization of this underutilized fish by-product. However, the present work represents a preliminary in vitro evaluation, and the peptides responsible for the observed activities were not identified. Furthermore, the study does not provide direct evidence of antihypertensive, hypoglycemic, nootropic, or other physiological effects in vivo. Therefore, additional studies are required to identify and characterize the bioactive peptides, evaluate their stability during digestion, determine their bioavailability and safety, and confirm their biological efficacy under in vivo conditions before any potential functional ingredient or nutraceutical application can be proposed.

## Figures and Tables

**Table 1:** Amino acid composition of protein from tilapia scales.

Amino Acids	Number of Residues/100
Asx	5.20
Thr	2.80
Ser	3.83
Glx	9.73
Pro	12.12
Gly	19.70
Ala	9.18
Cys	0.56
Val	2.10
Met	1.55
Ile	1.08
Leu	2.71
Tyr	1.26
Phe	2.56
His	1.16
Lys	3.13
Arg	7.77
OH-Pro	11.98
OH-Lys	1.50
Total	100

Glx = Glu + Gln; Asx = Asp + Asn.

**Table 2:** Biological activities of AP- and E-hydrolysates, expressed as mean ± SD. The IC_50_ values represent the amount of sample necessary to inhibit 50% of the enzymatic activity. Different letters in the same row (Student’s *t*-test) indicate significant differences between samples (*p* < 0.05).

Activity	AP-Hydrolysate	E-Hydrolysate
ABTS (mg ascorbic acid Equ /g)	18.76 ± 0.05 ^a^	18.75 ± 0.09 ^a^
FRAP (mEq Mohr salt/g)	40.85 ± 1.62 ^a^	34.14 ± 2.94 ^b^
Fe(II)-chelating activity (%Act/µg)	2.79 ± 0.71 ^a^	0.80± 0.09 ^b^
ACE (IC_50_ in µg/mL)	13.7 ± 0.2 ^a^	14.5 ± 4.8 ^a^
DPP-IV (IC_50_ in mg/mL)	0.66 ± 0.04 ^a^	0.69 ± 0.06 ^a^
PEP (IC_50_ in mg/mL)	0.72 ± 0.07 ^a^	0.63 ± 0.02 ^a^

**Table 3:** Inhibitory activities of tilapia scale hydrolysates after SGID in the gastric and intestinal phases. Data are presented as mean ± standard deviation (SD). Different letters within the same row indicate significant differences between hydrolysates (*p* < 0.05, Student’s *t*-test).

Activity	Phase	AP-Hydrolysate	E-Hydrolysate
ACE (IC_50_ µg/mL)	Gastric	6.0 ± 2.0 ^a^	5.5 ± 1.3 ^a^
	Intestinal	6.7 ± 1.4 ^a^	4.6 ± 0.1 ^b^
DPP-IV (IC_50_ mg/mL)	Gastric	1.06 ± 0.11 ^a^	1.63 ± 0.28 ^b^
	Intestinal	2.06 ± 0.18 ^b^	1.12 ± 0.12 ^a^
PEP (IC_50_ mg/mL)	Gastric	0.56 ± 0.21 ^a^	0.90 ± 0.04 ^b^
	Intestinal	2.61 ± 0.19 ^a^	1.89 ± 0.11 ^b^

## Data Availability

The original data presented in the study are openly available in DIGITAL.CSIC at http://hdl.handle.net/10261/430195.
